# External quality assessment of SARS-CoV-2 serology in European expert laboratories, April 2021

**DOI:** 10.2807/1560-7917.ES.2022.27.42.2101057

**Published:** 2022-10-20

**Authors:** Ramona Mögling, Francesca Colavita, Johan Reimerink, Angeliki Melidou, Katrin Leitmeyer, Maria Keramarou, Daniele Lapa, Massimo Francalancia, Jean-Luc Murk, Ann Vossen, Fabrizio Carletti, Boris Hogema, Adam Meijer, Liesbet Deprez, Antonino di Caro, Concetta Castilletti, Chantal BEM Reusken

**Affiliations:** 1 Centre for Infectious Disease Control, National Institute for Public Health and the Environment (RIVM), Bilthoven, the Netherlands; 2 National Institute for Infectious Diseases 'Lazzaro Spallanzani' IRCCS (INMI), Rome, Italy; 3 European Centre for Disease Prevention and Control (ECDC), Stockholm, Sweden; 4 Microvida, location St Elisabeth-Tweesteden Hospital, Tilburg, The Netherlands; 5 Leiden University Medical Center, Leiden, The Netherlands; 6 Sanquin Research, Amsterdam, The Netherlands; 7 European Commission, Joint Research Centre (JRC), Geel, Belgium; 8 Unicamillus, International Medical University, Rome, Italy; 9 IRCCS Sacro Cuore Don Calabria Hospital, Negrar di Valpolicella, Italy

**Keywords:** SARS-CoV-2, COVID-19, EQA, serology, antibodies, Europe

## Abstract

**Background:**

Countries worldwide are focusing to mitigate the ongoing SARS-CoV-2 pandemic by employing public health measures. Laboratories have a key role in the control of SARS-CoV-2 transmission. Serology for SARS-CoV-2 is of critical importance to support diagnosis, define the epidemiological framework and evaluate immune responses to natural infection and vaccine administration.

**Aim:**

The aim of this study was the assessment of the actual capability among laboratories involved in sero-epidemiological studies on COVID-19 in EU/EEA and EU enlargement countries to detect SARS-CoV-2 antibodies through an external quality assessment (EQA) based on proficiency testing.

**Methods:**

The EQA panels were composed of eight different, pooled human serum samples (all collected in 2020 before the vaccine roll-out), addressing sensitivity and specificity of detection. The panels and two EU human SARS-CoV-2 serological standards were sent to 56 laboratories in 30 countries.

**Results:**

The overall performance of laboratories within this EQA indicated a robust ability to establish past SARS-CoV-2 infections via detection of anti-SARS-CoV-2 antibodies, with 53 of 55 laboratories using at least one test that characterised all EQA samples correctly. IgM-specific test methods provided most incorrect sample characterisations (24/208), while test methods detecting total immunoglobulin (0/119) and neutralising antibodies (2/230) performed the best. The semiquantitative assays used by the EQA participants also showed a robust performance in relation to the standards.

**Conclusion:**

Our EQA showed a high capability across European reference laboratories for reliable diagnostics for SARS-CoV-2 antibody responses. Serological tests that provide robust and reliable detection of anti-SARS-CoV-2 antibodies are available.

**Key public health message**

**What did you want to address in this study?**

Robust serological test systems to detect antibodies against SARS-CoV-2 in human serum samples are important to establish whether past infections occurred and to assess the extent of immunity in the population. We want to enable laboratories and countries employing public health measures to assess the quality and robustness of different serological tests that are being used in European expert laboratories.

**What have we learnt from this study?**

The majority of laboratories (53 of 55) used at least one serological test that was able to characterise all the serum samples correctly. The performance of serological assays varied for different types of antibodies.

**What are the implications of your findings for public health?**

Our study showed a high capability across European reference laboratories for reliable diagnostics for SARS-CoV-2 antibody responses. Aside from assessing the total share of people with immunity in the population, reliable serological diagnostics are also important to guide public health actions by helping to estimate the proportion of asymptomatic cases.

## Introduction

Countries worldwide are focusing to mitigate the ongoing coronavirus disease (COVID-19) pandemic [1] by employing public health measures, including increasing vaccination roll-out and restriction of movements. Laboratories have a key role in the control of the transmission of severe acute respiratory syndrome coronavirus 2 (SARS-CoV-2) by detecting acute and previous infections with the virus in a reliable and timely fashion. While the detection of acute infections, typically done by real-time reverse transcription PCR (RT-PCR) or antigen (Ag) testing, can be used to stop transmission chains through isolation and quarantine measures [2], the detection of immunological markers for past SARS-CoV-2 infections and/or vaccinations against SARS-CoV-2 is used to estimate immunity [3] in both individuals and communities, thereby informing mitigation strategies. Most serological assays detect antibodies against SARS-CoV-2 as the main immunological marker. The emergence of SARS-CoV-2 variants in the spike protein represents one of the main concerns for the potential of immunological escape from the antibodies response.

The vast impact of the COVID-19 pandemic on public health, economies and societies drives the rapid development of numerous serological assays by laboratories and commercial entities [4]. These tests are not only based on different techniques, e.g. enzyme-linked immunosorbent assays (ELISA), chemiluminescence immunoassays (CLIA), lateral flow assays (LFA) and virus neutralisation tests (VNT), but also use different antigenic targets and are able to detect different types of anti-SARS-CoV-2 antibodies, e.g. total immunoglobulin (Ig), IgG, IgM, IgA and/or neutralising antibodies. Besides the fact that each laboratory needs to perform validation and evaluation studies before implementing a new test [5], it is crucial to assess the capability of the laboratory to perform the test through an external quality assessment (EQA) based on proficiency testing. Proficiency testing enables a comparison of the accuracy of different tests and the performance of different laboratories based on the same material [6-10]. 

Here, we describe the set-up and results of such an EQA of detection of SARS-CoV-2 antibodies among European expert laboratories that are members of the European Centre for Disease Prevention and Control (ECDC) COVID-19 and influenza laboratory networks, laboratories involved in sero-epidemiological studies on COVID-19 in the European Union and European Economic Area (EU/EEA) and EU-enlargement countries and/or members of the Emerging Viral Diseases-Expert Laboratory Network (EVD-LabNet). The proficiency panel comprised different isotypes of SARS-CoV-2 antibodies. In addition, laboratories also received two human SARS-CoV-2 serological standards produced and described by the Joint Research Centre (JRC), EURM-017 and EURM-018 [11,12], to assess the performance of semiquantitative assays used in different laboratories in comparison with reference material.

## Methods

### External quality assessment scheme organisation

In December 2020 and January 2021, European expert laboratories that are members of the ECDC COVID-19 and influenza laboratory networks, laboratories involved in sero-epidemiological studies on COVID-19 in EU/EEA and EU-enlargement countries and/or members of the Emerging Viral Diseases-Expert Laboratory Network (EVD-LabNet) were invited to participate this EQA study. Registration was closed on 2 February 2021.

Fifty-four laboratories received one EQA panel and two JRC standards between 22 February and 5 March 2021, one laboratory registered later and received the packages on 24 March 2021. The online submission form to submit EQA results was open until 8 April 2021.

### Panel composition

The EQA panels were composed of eight different, fully characterised, pooled human serum samples and addressed both sensitivity and specificity of detection. Five samples contained anti-SARS-CoV-2 antibodies, while three samples did not (Table 1). Anti-SARS-CoV-2 antibody-positive sera were collected from individuals with different severities of disease: mild, non-hospitalised cases (two samples that contained mixed sera from multiple patients) and severe, hospitalised cases (three samples that contained mixed sera from multiple patients). The sera from individuals with SARS-CoV-2 infection were a collection of residual routine diagnostic anonymised samples and not suitable to evaluate antibody waning. The median time from infection diagnosis to sample collection was 17.5 days (range: 5–92 days). The obtained serology results for the preparation of the EQA were not used for the clinical management of the patients. All sera from individuals with a SARS-CoV-2 infection were collected in 2020 before COVID-19 vaccine roll-out. The sera from individuals with acute cytomegalovirus (CMV) infection were collected before the emergence of SARS-CoV-2 at the end of 2019 and were anonymised. In addition, sera from individuals with an acute Epstein-Barr virus (EBV) infection were anonymised. The anti-SARS-CoV-2-negative sera were pre-pandemic residual sera from routine diagnostics provided by the National Institute of Infectious Diseases (INMI) in Rome, Italy and the National Institute for Public Health and the Environment (RIVM) in Bilthoven, the Netherlands. All panel sera, including the pre-pandemic sera, contained antibodies directed against the common cold coronaviruses (HCoV-OC43; HCoV-229E; HCoV-NL63; HCoV-HKU1). In addition to the EQA panel, all participants received two human SARS-CoV-2 serological standards produced and described by the JRC: EURM-017 and EURM-018 [11,12].

**Table 1:** SARS-CoV-2 serology external quality assessment panel composition and overall test results (n = 162) of participating laboratories (n = 55), EU/EEA, February–April 2021

Sample ID	Anti-SARS-CoV-2 antibodies present	Sample information	Correct results^a^		
			%	Number	Total
A	Yes	Hospitalised (IgA+/IgG+/IgM+)	98.8	160	162
B	Yes	Mild disease (IgA+/IgG+/IgM+)	98.7	157	159^b^
C	No	Negative pre-pandemic	95.0	153	161^b^
D	Yes	Hospitalised (IgA+/IgG+/IgM+)	99.4	161	162
E	No	Acute CMV+EBV infection	96.3	155	161^b^
F	No	Negative pre-pandemic	88.8	143	161^b^
G	Yes	Mild disease (IgA+/IgG+/low IgM+^c^)	96.3	156	162
H	Yes	Hospitalised (IgA+/IgG+/IgM+)	100.0	162	162
EURM-017	Yes	JRC standard (low IgA+^c^/IgG+/low IgM+^c^)	94.9	149	157
EURM-018	Yes	JRC standard (low IgA+^c^/IgG+/low IgM+^c^)	96.8	152	157

CMV: cytomegalovirus; EBV: Epstein–Barr virus; EU/EEA: European Union and European Economic Area; JRC: Joint Research Centre; SARS-CoV-2: severe acute respiratory syndrome coronavirus 2.

^a^ Correct results at test level.

^b^ Samples B, C, E, F and both standards were not tested with the maximum number (n = 162) of submitted tests. Laboratories did not indicate the reasons for not testing these panel entries.

^c^ Pooled serum samples contained anti-SARS-CoV-2 IgM and/or IgA antibodies but in low quantities.

### External quality assessment panel preparation

The freeze-dried equivalent of 0.2 mL pooled anonymised sera was prepared for each panel sample (Table 1). Samples were pooled to obtain sufficient volumes for uniform preparation of the total number of required panels. All pooled samples were heat-inactivated (56 °C for 30 min) and freeze-dried in 0.2 mL aliquots (FreeZone Benchtop Freeze Dryer, LABCONCO, United States (US)). Successful virus inactivation of panel samples was confirmed by the absence of viral growth in two consecutive cell culture passages. All samples were provided coded Sample A to Sample H, with no further identifying information given.

The standards EURM-017 and EURM-018 were produced and shipped by JRC. Product information on the materials is publicly available [11,12].

### Samples characterisation, testing instructions

Each sample of the EQA panel was extensively characterised by the World Health Organization (WHO) COVID-19 reference laboratories at INMI and RIVM, using a wide variety of serological tests (tests and outcomes of the panel characterisation are provided in Supplementary Table S1). Based on this characterisation, the final composition of the EQA panels was defined (Table 1). All samples of the EQA panel, including the anti-SARS-CoV-2-negative samples (C, E and F) were determined to contain antibodies against the four common human coronaviruses (HCoV-229E, HCoV-HKU1, HCoV-OC43, HCoV-NL63) by protein micro-array as a quantitative multiplex immunoassay [13].

The EQA panels were shipped at room temperature. The serological standards were shipped separately on dry ice. Laboratories received detailed reconstitution, testing and storage instructions with the panels, advising to centrifugate the lyophilised samples for 1 min at 3,000 rpm to sediment material which might stick to the cap, reconstitute the samples in 200 µL sterile water for ca 1 h, vortex the samples for ca 10 sec to resuspend the material properly, and centrifugate the samples to spin down the reconstituted materials to avoid contamination. Laboratories were informed that the EQA panel samples consisted of inactivated, non-infectious human sera, but no specific information was provided, i.e. positivity and negativity to anti-SARS-CoV-2.

The JRC standards were ready to use upon receipt. It was advised to store the material as follows: original material of the EQA panel at room temperature (acceptable range: −20 °C to +25 °C), and at 4 °C once the material was reconstituted; original material of the JRC standards frozen (acceptable range: −20 °C to −80 °C), and at 4 °C once the material was thawed. Laboratories were informed that the provided standards contained anti-SARS-CoV-2 antibodies, as they received the product information sheet alongside the EQA panel.

### Evaluation of results

The testing instructions provided a link to the online result submission platform. In the online submission form, laboratories were asked to give detailed information on each of the tests that they performed on the EQA panel and JRC standards, including the type of detectable antibodies. Since a the majority of serological tests only require small volumes, multiple different tests could be performed. For each method, the laboratories had to indicate whether they detected anti-SARS-CoV-2 antibodies in the EQA samples, the specific result of the test and how they interpreted the outcome of the test. Because laboratories had to submit their results per specific test used, the EQA outcomes could be analysed on individual test level, as well as on laboratory level (multiple tests per laboratory). There was no minimum or maximum limit for how many tests could be assessed with the EQA panels and submitted in the online submission form. 

**Figure 1:**
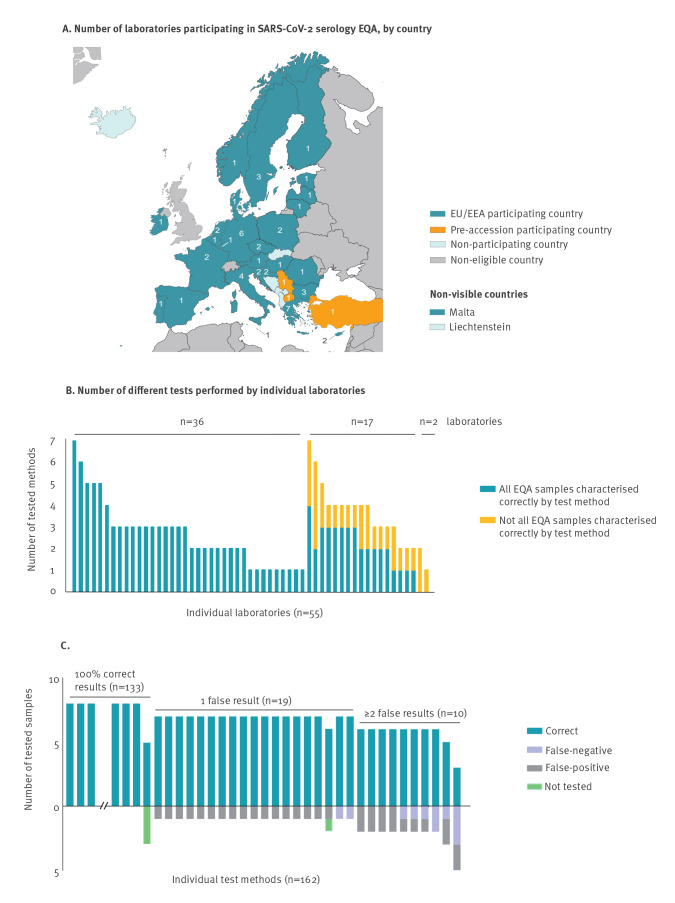
Number of expert laboratories participating in external quality assessment, per country and overall laboratory (n = 55) and test (n = 162) performance, EU/EEA, February–April 2021

EEA: European Economic Area; EQA: external quality assessment; EU: European Union.

For the purposes of this EQA, the panel outcomes with an individual test submitted by a laboratory were referred to as a ‘test’, i.e. in total, results of 162 tests were submitted by 55 laboratories (for instance, two laboratories used seven tests to characterise the EQA samples, 16 used three tests, 10 used one test only) (Figure 1). Tests that were used by multiple laboratories that were either the same commercial test or had the same principle (e.g. VNT) were referred to as ‘assays’, i.e. in total 53 commercial and in-house assays (Supplementary Table S2 lists the commercial and in-house tests assessed by EQA participants) were used by 55 laboratories resulting in a submission of results of 162 tests.

### Statistics

Data were collected and analysed in Microsoft Excel (Microsoft Corp., Bellingham, US) and GraphPad Prism 9 software for Windows version 9.1.0 (GraphPad Software, San Diego, US). Performance of specific tests was analysed by comparison of the amount of correct vs false results, either grouped by assay type or by isotypes of detectable antibodies, using two-sided Yates’ corrected chi-squared test. Results with a p value ≤ 0.05 were considered statistically significant. Furthermore, Spearman’s rank correlation test was used to assess correlation of EQA performance as fraction of correct results, with sample input volume as specified by the EQA participants.

## Results

### External quality assessment participation and overall laboratory and test performance

Fifty-six laboratories registered to participate in the EQA. In total, 55 laboratories from 30 countries, namely 27 of 30 EU/EEA countries and three of seven EU pre-accession countries, reported individual panel results representing 162 tests (Figure 1A), while one laboratory did not submit any results. The number of different tests assessed per laboratory varied between 1 and 7 (Figure 1B).

Thirty-six of 55 laboratories characterised all EQA samples correctly with all tests they assessed, while 17 additional laboratories used at least one test that identified all EQA samples correctly (Figure 1B). Only two laboratories could not identify all samples correctly (Figure 1B). On test level, 82.1% of the submitted tests (133/162) detected all tested samples correctly. While the outcome for the samples of pooled sera from hospitalised patients and for one sample of the pooled sera from mild patients (A, D, H and B) ranged between 98.7% and 100.0% correct results, samples C (negative pre-pandemic), E (acute CMV+EBV infection) and G (mild COVID-19, A+/G+/M+(low)) showed slightly lower proportions for correct identification ranging from 95.0% to 96.3% (Table 1). The sample with the least correct results, 88.8%, was sample F (negative pre-pandemic, Table 1). The correct identification proportion for the JRC standards was 94.9% for EURM-017 and 96.8% for EURM-018 (Table 1).

### Assay performance

Overall, false-negative results occurred less frequently than false-positive results (Figure 1C, Table 2). Of the 161 tests, 135 (83.9%) characterised all specificity samples (C, E, F) correctly. Furthermore, we analysed assays separately for each antibody isotype they could detect when laboratories submitted these individual results. Assays for which no individual isotype results were submitted were grouped as ‘IgM/IgG’ and ‘IgM/IgA’ (Table 2). IgM-specific tests performed least accurately and were significantly worse at identifying samples correctly than tests detecting total immunoglobulin (Table 2). For each group, IgA, IgG or IgM, multiple assays gave at least one false outcome; respectively, two of six, seven of 32 and eight of 14 assays had at least one false result.

**Table 2:** Performance of assays in external quality assessment, grouped by SARS-CoV-2 antibody isotype detection, EU/EEA, February–April 2021 (n = 171)

Antibody type(s) detectable by assay	Number of submitted tests per antibody type^a^	Number of different assays per antibody type^a^	Number of false-negative tests					Number of false-positive tests			Performance compared with total Ig^b^ p value	
			Sample A – hospitalised	Sample D – hospitalised	Sample H – hospitalised	Sample B – mild	Sample G – mild	Sample C – negative	Sample F – negative	Sample E – negative	False-negative results	False-positive results
			IgA+/IgG+/IgM+	IgA+/IgG+/IgM+	IgA+/IgG+/IgM+	IgA+/IgG+/IgM+	IgA+/IgG+/low IgM+	Pre-pandemic	Pre-pandemic	Acute CMV+EBV		
Total Ig	15	3	0	0	0	0	0	0	0	0	NA	NA
IgA	19	6	1	1	0	0	1	2	2	1	0.3394	0.1151
IgG	78	32	1	0	0	1	1	2	4	4	0.9741	0.3243
IgM	26	14	0	0	2	1	6	3	11	1	0.0499	0.0043
IgM/IgG**^c^**	1	1	0	0	0	0	0	0	0	0	NA	NA
IgM/IgA**^c^**	3	1	0	0	0	0	0	0	0	0	NA	NA
Neutralising antibodies	29	8	0	0	0	0	0	1	1	0	NA	0.7846

CMV: cytomegalovirus; EBV: Epstein–Barr virus; EEA: European Economic Area; EU: European Union; NA: not applicable; SARS-CoV-2: severe acute respiratory syndrome coronavirus 2.

**^a^** Assays that are able to detect multiple antibody types, and for which individual results for the respective antibody types were submitted, were analysed separately for each antibody type in this table.

**^b^** Performance of antibody type specific tests was compared with performance of total Ig tests using two-sided Yates’ corrected chi-squared test. Antibody-specific tests that performed significantly worse than total immunoglobulin tests are underlined.

**^c^** Combinations of multiple antibody types are possible for some tests. For assays of the groups ‘IgM/IgG’ and ‘IgM/IgA’, EQA participants did not submit the individual results for the respective antibody types.

In total, 41 commercial assays and 12 in-house assays were used by the EQA participants (listed in Supplementary Table S2). Overall, 3.5% (37/1,058) of reported results using commercial assays and 2.6% (6/231) using in-house assays were incorrect. Of all 162 tests used by participants, 75 (46.3%) were ELISA-based, 43 (26.5%) were CLIA/chemiluminescent microparticle immunoassay (CMIA)/electrochemiluminescent immunoassay (ECLIA) tests and 22 (13.6%) VNTs. The remaining 22 (13.6%) included, among other types of tests, LFA, plaque reduction neutralisation test (PRNT), protein micro-array and immunofluorescence assay/enzyme-linked fluorescence assay.

Among those assays performed by three or more EQA participants, two in-house assays (VNT, PRNT) and six commercial assays (Abbott – SARS-CoV-2 IgG II Quant, Beijing Wantai Biological – SARS-CoV-2 Ab ELISA, Euroimmun – Anti-SARS-CoV-2 QuantiVac ELISA IgG, Roche – Elecsys Anti-SARS-CoV-2 S, Vircell microbiologists – COVID-19 ELISA IgM+IgA and GenScript - SARS-CoV-2 Surrogate Virus Neutralisation Test Kit) correctly characterised all EQA samples in all laboratories that assessed these assays (Table 3). None of the assays performed significantly better than the group ‘Other’ (i.e. all tests that were used by fewer than three laboratories) considering two-sided Yates’ corrected chi-squared test. One test (Euroimmun – Anti-SARS-CoV-2 NCP ELISA IgM) performed significantly worse than ‘Other’ (p value < 0.0001) (Table 3).

**Table 3:** Performance of different SARS-CoV-2 assays that were used by three or more laboratories, external quality assessment, EU/EEA, February–April 2021 (n = 18)

Method details		Method type	Antibody type	Unit	Assay performance (samples characterised correctly/samples tested)								EQA performance		
					Sample A – hospitalised	Sample D – hospitalised	Sample H – hospitalised	Sample B – mild	Sample G – mild	Sample C – negative	Sample F – negative	Sample E – negative	Total correct (samples characterised correctly/samples tested)	False-negative results	False-positive results
					IgA+/IgG+/IgM+	IgA+/IgG+/IgM+	IgA+/IgG+/IgM+	IgA+/IgG+/IgM+	IgA+/IgG+/low IgM+	Pre-pandemic	Pre-pandemic	Acute CMV+EBV			
Abbott	SARS-CoV-2 IgG	CLIA/CMIA	IgG	Index (S/C)	11/12	12/12	12/12	11/12	11/12	12/12	12/12	12/12	93/96	3	0
Abbott	SARS-CoV-2 IgG II Quant	CLIA/CMIA	IgG	AU/mL^b^	9/9	9/9	9/9	9/9	9/9	9/9	9/9	9/9	72/72	0	0
Abbott	SARS-CoV-2 IgM	CLIA/CMIA	IgM	Index (S/C)	6/6	6/6	6/6	5/6	6/6	5/6	6/6	6/6	46/48	1	1
Beijing Wantai Biological	SARS-CoV-2 Ab ELISA	ELISA	Total Ig	Ratio	10/10	10/10	10/10	9/9	10/10	10/10	10/10	10/10	79/79	0	0
Beijing Wantai Biological	SARS-CoV-2 IgM ELISA	ELISA	IgM	Ratio	3/3	3/3	3/3	3/3	3/3	3/3	1/3	3/3	22/24	0	2
Diasorin Inc.	LIAISON SARS-CoV-2 S1/S2 IgG	CLIA/CMIA	IgG	AU/mL^b^	5/5	5/5	5/5	4/4	5/5	4/4	4/4	2/4	34/36	0	2
Euroimmun	Anti-SARS-CoV-2 ELISA IgA	ELISA	IgA	Ratio	14/14	14/14	14/14	14/14	14/14	13/14	12/14	14/14	109/112	0	3
Euroimmun	Anti-SARS-CoV-2 ELISA IgG	ELISA	IgG	Ratio	17/17	17/17	17/17	17/17	17/17	17/17	15/17	17/17	134/136	0	2
Euroimmun	Anti-SARS-CoV-2 NCP ELISA IgG	ELISA	IgG	Ratio	3/3	3/3	3/3	3/3	3/3	3/3	2/3	3/3	23/24	0	1
Euroimmun	Anti-SARS-CoV-2 NCP ELISA IgM	ELISA	IgM	Ratio	5/5	5/5	5/5	5/5	1/5	4/5	0/5	5/5	30/40	4	6
Euroimmun	Anti-SARS-CoV-2 QuantiVac ELISA IgG	ELISA	IgG	RU/mL^b^	4/4	4/4	4/4	4/4	4/4	4/4	4/4	4/4	32/32	0	0
GenScript	cPass SARS CoV-2 Neutralisation Antibody Detection Kit	sVNT	Neutralising Ab	% of inhibition	3/3	3/3	3/3	3/3	3/3	3/3	2/3	3/3	23/24	0	1
GenScript	SARS-CoV-2 Surrogate Virus Neutralisation Test Kit	sVNT	Neutralising Ab	% of inhibition	5/5	5/5	5/5	5/5	5/5	5/5	5/5	5/5	40/40	0	0
In-house	NA	ELISA	IgA, IgG, IgM	Index	4/4	4/4	4/4	4/4	4/4	2/4	2/4	4/4	28/32	0	4
In-house	NA	PRNT	Neutralising Ab	Titre	4/4	4/4	4/4	4/4	4/4	4/4	4/4	4/4	32/32	0	0
In-house	NA	(s)VNT	Neutralising Ab	Titre	14/14	14/14	14/14	13/13	14/14	14/14	14/14	14/14	111/111	0	0
Other^a^	NA	Various	Various	Various	40/41	40/41	41/41	41/41	40/41	38/41	38/41	37/41	315/328	3	10
Roche	Elecsys Anti-SARS-CoV-2 S	CLIA/CMIA	Total Ig	U/mL^b^	3/3	3/3	3/3	3/3	3/3	3/3	3/3	3/3	24/24	0	0
Vircell microbiologists	COVID-19 ELISA IgM + IgA	ELISA	IgA + IgM	Index	3/3	3/3	3/3	3/3	3/3	3/3	3/3	3/3	24/24	0	0

Ab: antibody; AU: arbitrary unit; CLIA: chemiluminescence immunoassay; CMIA: chemiluminescent microparticle immunoassay; CMV: cytomegalovirus; COVID-19: coronavirus disease; EBV: Epstein–Barr virus; EQA: external quality assessment; EEA: European Economic Area; ELISA: enzyme-linked immunosorbent assay; EU: European Union; Ig: immunoglobulin; NA: not applicable; PRNT: plaque reduction neutralisation test; RU: relative units; U: units; SARS-CoV-2: severe acute respiratory syndrome coronavirus 2; (s)VNT: (surrogate) virus neutralisation test.

^a^ The category ‘other’ comprises all tests that were used by less than three laboratories.

### Qualitative and semiquantitative results in relation to Joint Research Centre standards

Four qualitative assays in this EQA had been previously used to characterise the JRC standards as positive, both EURM-017 [11] and EURM-018 [12]: Genscript (cPass SARS-CoV-2 Neutralisation Antibody detection kit), Roche (Elecsys Anti-SARS-CoV-2), Abbott (SARS-CoV-2 IgG) and Abbott (SARS-CoV-2 IgM). In this EQA, Genscript and Roche scored all samples correctly, while the assays of Abbott SARS-CoV-2 IgG and SARS-CoV-2 IgM each scored one standard incorrectly (EURM-018) as negative.

**Figure 2:**
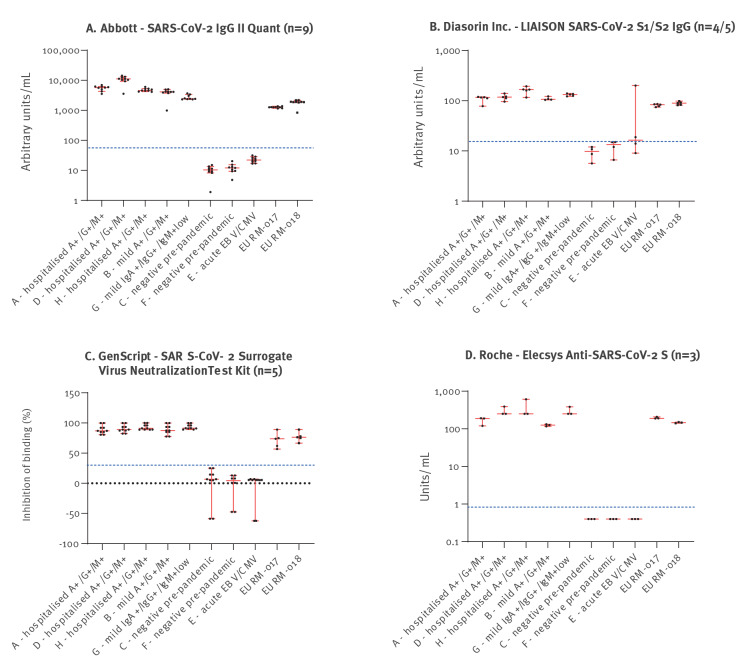
Reported numerical results for EQA and JRC standards (EURM-017 and EURM-018), semiquantitative SARS-CoV-2 assays used by at least three laboratories, EU/EEA, February–April 2021 (n = 22)

EEA: European Economic Area; EQA: external quality assessment; EU: European Union; JRC: Joint Research Council; SARS-CoV-2: severe acute respiratory syndrome coronavirus 2.

Laboratories were asked to specify the exact (numerical) results of each test they used to assess how semiquantitative assays performed across different laboratories in comparison to the reference product information available for the two JRC standards. Figure 2 and Table 4 show an overview of the numerical results for all semiquantitative specific assays that were used by three or more laboratories. Notably, results of three of the semiquantitative assays, namely by Abbott (SARS-CoV-2 IgG II Quant), GenScript (cPass SARS-CoV-2 Surrogate Virus Neutralisation Test Kit) and Roche (Elecsys Anti-SARS-CoV-2 S) characterised all EQA samples correctly; their results for the JRC standards corresponded to previous JRC characterisations (Table 4). Absolute titres obtained with the various virus neutralisation assays varied between laboratories (range: 12- to 115-fold differences).

In panel B, n = 4/5 indicates that one laboratory only tested five of eight EQA samples with this test method. Dotted blue lines indicate method specific cut-off values for positivity as indicated by EQA participants and manufacturers’ instructions. Red lines show median and 95.0% confidence interval.

**Table 4:** Results for JRC standards EURM-017 and EURM-018, semiquantitative SARS-CoV-2 assays used by three or more laboratories^a^ in this EQA and previously available product information for the reference material, EU/EEA, February–April 2021 (n = 22)

Method details		EQA participants		JRC reference product information^b^	
		EURM-017	EURM-018	EURM-017	EURM-018
Abbott - SARS-CoV-2 IgG II Quant, n = 9	Median	1,271 AU/mL	1,914 AU/mL	NA	NA
	95.0% CI of median lower confidence limit	1,174 AU/mL	1,815 AU/mL	NA	NA
	95.0% CI of median upper confidence limit	1,367 AU/mL	2,157 AU/mL	NA	NA
	Mean	1,271 AU/mL	1,836 AU/mL	1,155 AU/mL	1,797 AU/mL
	Standard deviation	74 AU/mL	397 AU/mL	NA	NA
Diasorin Inc. - LIAISON SARS-CoV-2 S1/S2 IgG, n = 5^c^	Median	84 AU/mL	89 AU/mL	NA	NA
	95.0% CI of median lower confidence limit	74 AU/mL	82 AU/mL	NA	NA
	95.0% CI of median upper confidence limit	86 AU/mL	98 AU/mL	NA	NA
	Mean	81 AU/mL	89 AU/mL	NA	NA
	Standard deviation	5 AU/mL	7 AU/mL	NA	NA
GenScript - SARS-CoV-2 Surrogate Virus Neutralisation Test Kit, n = 5	Median	73.8% inhibition	76.4% inhibition	NA	NA
	95.0% CI of median lower confidence limit	56.7% inhibition	66.6% inhibition	NA	NA
	95.0% CI of median upper confidence limit	89.0% inhibition	89.0% inhibition	NA	NA
	Mean	71.3% inhibition	77.0% inhibition	73.8% inhibition^d^	76.4% inhibition^d^
	Standard deviation	12.6% inhibition	8.0% inhibition	NA	NA
Roche - Elecsys Anti-SARS-CoV-2 S, n = 3	Median	190 U/mL	146 U/mL	NA	NA
	95.0% CI of median lower confidence limit	188 U/mL	141 U/mL	NA	NA
	95.0% CI of median upper confidence limit	208 U/mL	151 U/mL	NA	NA
	Mean	195 U/mL	146 U/mL	199 U/mL	154 U/mL
	Standard deviation	11 U/mL	5 U/mL	NA	NA

AU: arbitrary unit; CI: confidence interval; EEA: European Economic Area; EQA: external quality assessment; EU: European Union; JRC: Joint Research Council; NA: not available; SARS-CoV-2: severe acute respiratory syndrome coronavirus 2; U: Unit.

**^a^** One additional semiquantitative assay, namely Euroimmun (Anti-SARS-CoV-2 QuantiVac ELISA IgG, n = 4) was used by more than three laboratories. However, the results could not be included for this analysis, as not all laboratories submitted results in units as prescribed by the manufacturer.

**^b^** Reference material product information published by JRC for JRC standards EURM-017 [11] and EURM-018 [12].

^c^ One laboratory only tested five of eight EQA samples with this test method.

**^d^** Information not publicly available, direct communication by JRC.

### Influence of various parameters on the performance of the external quality assessment

The vast majority of laboratories used the advised volume of 200 µL sterile water to reconstitute the EQA samples (51/55 laboratories) as well as the storage advice for both the EQA panels (54/55 laboratories) and the JRC standards (50/55 laboratories). We did not see any influence of these parameters on test performance. Although sample input volume for the different tests ranged from 1 µL to 170 µL, it did not correlate with the fraction of correct results by test (p = 0.3251; Spearman correlation test).

Considering antibodies directed to different antigenic proteins targeted in the serological assays, the majority of the assays used (n = 27) was based on the spike (S) protein, including recombinant full S or its specific domains (S1, S2 or receptor-binding domain). Fifteen assays used both N and S proteins, while six targeted the anti-N response only. In addition, 18 assays used whole live virus isolates for VNT/PRNT, while for three in-house assays, the viral antigen was not reported. No influence of the target protein on test results was observed.

## Discussion

The overall performance of laboratories within this EQA indicated a robust ability to establish whether past SARS-CoV-2 infections occurred via detection of anti-SARS-CoV-2 antibodies. All except two laboratories used at least one assay that identified all samples correctly as SARS-CoV-2 antibody positive or negative.

IgM-specific assays showed the most incorrect characterisations of the EQA samples. In particular, sample G (IgA+/IgG+/IgM+(low)) was missed by four of five laboratories that used the same commercial IgM-only assay (Euroimmun – Anti-SARS-CoV-2 NCP ELISA IgM) and by two additional assays that should have been able to detect the presence of IgM antibodies based on their technical specifications (AAZ - COVID-PRESTO TROD IgG/IgM; Hangzhou Biotest Biotech - RightSign COVID-19 IgG/IgM Rapid Test Cassette). The remaining 20 laboratories were able to identify sample G correctly using 11 different assays. Indeed, this sample was characterised by the two reference laboratories to have IgM levels below the detection limit of some assays (find the detailed results listed in Supplementary Table S1). Furthermore, eight of 14 IgM-specific assays resulted in one or more false-positive test results. Several studies have shown that in general, IgM assays have a lower sensitivity and specificity than IgG or total IgG assays [14-16]. Hence it is recommended to perform sero-diagnostics on multiple isotypes simultaneously to improve the specifics of the overall diagnosis. In addition, although specific IgM antibodies can be detected as early as 4 days after infection and they can help to define the early antibody response, SARS-CoV-2 infection may trigger unconventional antibody responses, with cases developing IgG before IgM or others with no IgM [17-19]. Indeed, the majority of laboratories (45/55) used more than one serological test to assess the samples. In case of result discrepancies between IgM-specific tests and tests detecting other types of immunoglobulins, laboratories should consider the timing of a potential infection, but also the higher unreliability of IgM-specific tests. Overall, test methods detecting total immunoglobulin, neutralising antibodies or IgG performed better than test methods that are IgA- or IgM-specific. Test method performance was different neither for commercial and in-house assays, nor for a specific test principle, e.g. ELISA, (s)VNT, PRNT or CLIA/CMIA/ECLIA. This was also shown by the variety of test methods that characterised 100.0% of tested samples correctly (Table 3).

Correct characterisation of the three specificity samples (C, E and F) was more problematic than of the sensitivity samples. Variable test performance for specificity samples has been reported before [20,21]. A likely explanation is potential cross-reactivity with antibodies raised by previous infection with other human coronaviruses causing the common cold [13], as all samples in the proficiency panel of this EQA also contained antibodies against all other seasonally circulating human coronaviruses (Supplementary Table S1). If this was the case, specificity test performance would probably be further reduced in periods of high prevalence of co-circulating seasonal coronaviruses.

As expected, the absolute titres obtained with the various virus neutralisation assays varied between laboratories as underlying protocols can vary extensively (e.g. whole viruses or pseudotype viruses, number of median tissue culture infectious dose units, incubation period and temperature, cell lines, cut-off, read-out) and this gold standard serology method is mostly used as a research tool in the virology field. A recent European study showed that indeed a substantial heterogeneity exists in neutralising antibody testing approaches, resulting in almost 100-fold differences in raw neutralising titres. However, a direct comparison was possible through harmonisation by the use of a standard defined in IU/mL, which reduced the inter-laboratory variability ca 10-fold [22]. Another study showed that the inter-laboratory variation for neutralisation tests was reduced more than 50-fold when assay outcomes were reported relative to the same (WHO) standard [23]. In our study, the results of the VNT could not be compared through normalisation as the JRC standards were defined with a titre range for neutralisation tests and not with a defined IU/mL as in the WHO standard [24].

The characterisation of the JRC standards with the semiquantitative binding assays by the EQA participants was in range with the standards product information, indicating a robust performance of these semiquantitative assays across different laboratories. The qualitative assays that were used to define the JRC standards performed similarly in the EQA as well.

Notably, all samples used in this study were obtained from patients with natural SARS-CoV-2 infections. We did not include samples from vaccinated individuals. For future sero-epidemiological surveillance studies, the capacity of serological tests to differentiate between antibodies derived from natural infections and antibodies induced by vaccination is desirable. This can be achieved by using serological tests with different antigenic targets, i.e. anti-N (natural infection only) and anti-S detection, to determine the level of natural immunity in the study population and assess the risk of vaccination not being sufficient to protect against a potentially increasing burden on the healthcare system. EQA programmes including also sera from vaccinated people would be of great interest to expand the evaluation of the accuracy of different tests during the next phases of the pandemic when vaccination campaigns are consolidated in most countries worldwide.

Proficiency testing in EQA schemes allows to compare the accuracy of different tests and the performance of different laboratories based on the same material. Although the number of study participants (n = 55) and moreover the total number of performed tests (n = 162) was sufficient to gain valuable insights into the quality of serological SARS-CoV-2 assays, the abundance, availability and variety of different SARS-CoV-2 assays used by the participants, limits the number of results obtained per specific assay by different laboratories and therefore the statistical power and confidence in the results. It would be desirable to perform additional follow-up studies. The wide variety in tests used and general lack of routine incorporation of international standards in SARS-CoV-2 serology prevents comparisons of immune responses across laboratories, thereby hampering standardised cross-border sero-epidemiological surveillance and the wide implementation of immunoassays to identify correlates of protection against SARS-CoV-2, e.g. in the context of vaccination policies or the EU Digital COVID Certificate Regulation [25].

Nevertheless, proficiency testing is important for individual laboratories. The results of an EQA allow laboratories to identify potential problems and improve the reliability of their diagnosis. Interestingly, one laboratory repeated their tests upon receipt of their EQA results which included three false negative results using two different assays. In this second attempt, the results were correct. This laboratory indicated insufficient homogenisation of the samples as a potential explanation for the observed discrepancies.

## Conclusion

A general capability for reliable, harmonised and standardised characterisation of the main immunological markers of a previous SARS-CoV-2 infection or vaccination against SARS-CoV-2 is crucial to increase the overall utility of serology testing. Potential inconsistent test performance and the absence of a rationale to use quantitative antibody assays due to the, yet, undetermined correlates of protection of antibody levels, currently limits the overall usefulness of serology. Participation in EQAs may help to improve implementation of diagnostic tests. Our EQA showed a high capability across European reference laboratories for reliable diagnostics for SARS-CoV-2 antibody responses. Serological tests that provide robust and reliable detection of anti-SARS-CoV-2 antibodies are available. However, the use of standards is necessary for meaningful quantitative measurements to increase the overall use of serology testing, including the evaluation of antibody waning and possible reactivity against more relevant virus variants with immunological escape potential in the prospective of a more ‘personalised’ approach to vaccination strategies. This is particularly relevant given that serology is no longer restricted to reference laboratories. Numerous laboratories non specialised in microbiology are performing serological tests using a wide panel of commercial assays. In order to maintain serological test quality, it would also be advisable to implement EQAs at national level, including schemes to evaluate and distinguish natural immunity and vaccination response. 

## Supplementary Data

171000 Supplement
