# Erratum for Euro Surveill. 2021;27(42)

**DOI:** 10.2807/1560-7917.ES.2022.27.43.221027e

**Published:** 2022-10-27

**Authors:** Eurosurveillance editorial team

**Affiliations:** 1 European Centre for Disease Prevention and Control (ECDC), Stockholm, Sweden

A correction was made in the article ‘External quality assessment of SARS-CoV-2 serology in European expert laboratories, April 2021’ by Mögling et al. published on 20 October 2022. A superscript “b” was included in some of the cells in the Unit column in Table 3 in the originally published version. These superscripts had no associated footnote and were removed on 25 October 2022. We apologise for this oversight.

