# Supplementary material for: External quality assessment of SARS-CoV-2 serology in European expert laboratories, April 2021
Source: Euro Surveill. 2022 Oct 20;27(42):2101057. doi: 10.2807/1560-7917.ES.2022.27.42.2101057 (PMC9585882; doi:10.2807/1560-7917.ES.2022.27.42.2101057)
Supplement: Supplement [file 21-01057_REUSKEN_Supplement.pdf]

Supplemental material - EQA Panel characteristics and assays performed by the participating laboratories

This supplementary material is hosted by Eurosurveillance as supporting information alongside the article ‘External Quality Assessment of SARS-CoV-2 serology in European Expert Laboratories: April 2021‘, on behalf of the authors, who remain responsible for the accuracy and appropriateness of the content. The same standards for ethics, copyright, attributions and permissions as for the article apply. Supplements are not edited by Eurosurveillance and the journal is not responsible for the maintenance of any links or email addresses provided therein.

Table S1. Tests and outcomes of pre-distribution panel characterisation of EQA panels by WHO COVID-19 reference laboratories, INMI and RIVM.

WHO COVID-19 reference laboratory	Type of test	Type of antibody	Test information	Unit	cut-off value	A - hospitalised<br>A+/G+/M+	D - hospitalised<br>A+/G+/M+	H - hospitalised<br>A+/G+/M+	B - mild<br>A+/G+/M+	G - mild<br>A+/G+/M+^{low}	C - negative<br>pre-pandemic	F - negative<br>pre-pandemic	E -<br>acute EBV/CMV infection
INMI	ELISA	IgA	DIESSE Diagnostica Senese Spa - ENZY-WELL SARS-CoV-2 IgA	ratio	≥1.1	9.6	3.4	4	4.8	7.2	0.6	0.6	1.4
	ELISA	IgM	DIESSE Diagnostica Senese Spa - ENZY-WELL SARS-CoV-2 IgM	ratio	≥1.1	2.5	3.2	1.2	3.4	0.6	0.3	0.4	0.5
	ELISA	IgG	DIESSE Diagnostica Senese Spa - ENZY-WELL SARS-CoV-2 IgG	ratio	≥1.1	7.1	8.1	7.6	9.6	5.2	0.5	0.3	1
	CLIA	IgG	Diasorin Inc. - LIAISON SARS-CoV-2 S1/S2 IgG	AU/ml	≥14	122	125	182	114	108	7.71	8.99	24.7
	CMIA	IgM	Abbott - SARS-CoV-2 IgG II Quant	AU/ml	≥50	7215.4	13166.8	5215.7	4844	2837.9	13.9	6.9	14.6
	CMIA	IgG	Abbott - SARS-CoV-2 anti-N IgG (CMIA)	ratio	≥1.4	5.46	7.43	5.97	6.68	4.48	0.23	0.37	0.63
	VNT90	neutralising antibodies	in-house - virus microneutralisation (VNT90)	titer	≥10	320	160	80	>640	40	<10	<10	<10
RIVM	ELISA	Total Ig	Beijing Wantai Biological - SARS-CoV-2 Ab ELISA	ratio	≥1	22.47	25.34	23.02	21.01	31.36	0.01	0.01	0.01
	ELISA	IgM	Beijing Wantai Biological - SARS-CoV-2 IgM ELISA	ratio	≥1	21.31	9.29	6.53	26.50	2.30	0.09	0.43	0.00
	ELISA	IgA	Euroimmun - Anti-SARS-CoV-2 ELISA IgA	ratio	≥1.1	11.10	10.12	10.42	11.33	8.85	0.44	0.64	0.46
	ELISA	IgG	Euroimmun - Anti-SARS-CoV-2 ELISA IgG	ratio	≥1.1	8.10	7.86	8.64	7.38	7.21	0.27	0.69	0.14
	sVNT	neutralising antibodies	GenScript - SARS-CoV-2 Surrogate Virus Neutralisation Test Kit	Percentage of inhibition	30.0	99.99	99.99	100.00	99.99	100.00	5.18	0.81	5.59

Table S1 Continued. Tests and outcomes of pre-distribution panel characterisation of EQA panels by WHO COVID-19 reference laboratories, INMI and RIVM.

WHO COVID-19 reference laboratory	Type of test	Type of antibody	Test information	Unit	cut-off value	A - hospitalised A+/G+/M+	D - hospitalised A+/G+/M+	H - hospitalised A+/G+/M+	B - mild A+/G+/M+	G - mild A+/G+/M+low	C - negative pre-pandemic	F - negative pre-pandemic	E - acute EBV/CMV infection
	VNT50	neutralising antibodies	in-house - virus microneutralisation (VNT50)	titer	≥10	>640	400	480	>640	140	<10	<10	<10
	Micro-array	IgG	in-house - protein micro-array SARS-CoV-2-S-T	titer	≥10	>14580	8800	5016	6908	2754	10	10	10
			in-house - protein micro-array SARS-CoV-2-S1	titer	≥10	3470	2330	2398	1808	1397	25	51	10
			in-house - protein micro-array SARS-CoV-2-N	titer	≥10	10206	3532	4006	>14580	276	10	40	10
			in-house - protein micro-array 229E-S-T	titer	≥10	1603	862	816	1382	1460	1227	1005	768
			in-house - protein micro-array HKU1-S-T	titer	≥10	9775	4202	4198	5271	1716	570	487	547
			in-house - protein micro-array HKU1-S1	titer	≥10	874	458	457	356	490	572	479	405
			in-house - protein micro-array OC43-S-T	titer	≥10	7101	6496	3783	3740	1786	1272	1019	730
			in-house - protein micro-array OC43-S1	titer	≥10	656	2114	468	778	888	1408	1278	1016
			in-house - protein micro-array NL63-S-T	titer	≥10	2302	1640	1597	1753	1823	1264	1075	1247
			in-house - protein micro-array NL63-S1	titer	≥10	387	418	195	339	395	514	508	504

Table S2. Commercial and in-house tests assessed by EQA participants.

Type of test	Test #	Test information	No. laboratories
commercial	1	Abbott - SARS-CoV- IgM (CMIA)	6
commercial	2	Abbott - SARS-CoV-2 IgG (CMIA)	12
commercial	3	Abbott - SARS-CoV-2 IgG II Quant	9
commercial	4	AESKU Diagnostics - AESKULISA SARS-CoV-2 S1 IgG	1
commercial	5	AESKU Diagnostics - AESKULISA SARS-CoV-2 S1 IgM	1
commercial	6	AMP diagnostics - AMP Rapid Test SARS-CoV-2 IgG/IgM	1
commercial	7	AAZ - COVID-PRESTO TROD IgG/IgM	1
commercial	8	Beckman Coulter - Access SARS-CoV-2 IgG II Assay	1
commercial	9	Beijing Wantai Biological - SARS-CoV-2 Ab ELISA	10
commercial	10	Beijing Wantai Biological - SARS-CoV-2 IgM ELISA	3
commercial	11	BioMerieux - VIDAS SARS-COV-2 IgG	2
commercial	12	BioMerieux - VIDAS SARS-COV-2 IgM	2
commercial	13	Diasorin Inc. - LIAISON SARS-CoV-2 S1/S2 IgG	5
commercial	14	Diasorin Inc. - LIAISON SARS-CoV-2 TrimericS IgG	2
commercial	15	Diasorin Inc. - LIAISON SARS-CoV-2 IgM	1
commercial	16	Euroimmun - Anti-SARS-CoV-2 ELISA IgA	14
commercial	17	Euroimmun - Anti-SARS-CoV-2 ELISA IgG	17
commercial	18	Euroimmun - Anti-SARS-CoV-2 NCP ELISA IgG	3
commercial	19	Euroimmun - Anti-SARS-CoV-2 NCP ELISA IgM	5
commercial	20	Euroimmun - Anti-SARS-CoV-2 QuantiVac ELISA IgG	4
commercial	21	Eurospital Diagnostic - ELISA SARS CoV-2 IgG	1
commercial	22	Generic assays - ELISA, GA CoV-2 IgG	1
commercial	23	Generic assays - ELISA, GA CoV-2 IgM	1
commercial	24	GenScript - cPass SARS CoV-2 Neutralisation Antibody Detection Kit	3
commercial	25	GenScript - SARS-CoV-2 Surrogate Virus Neutralisation Test Kit	5
commercial	26	Hangzhou Biotest Biotech - RightSign COVID-19 IgG/IgM Rapid Test Cassette	1

Table S2 continued. Commercial and in-house tests assessed by EQA participants.

Type of test	Test #	Test information	No. laboratories
commercial	27	ImmunoDiagnostics - SARS-CoV-2 NP IgG ELISA Kit	1
commercial	28	ImmunoDiagnostics - SARS-CoV-2 S1 IgG ELISA Kit	1
commercial	29	Maccura Biotechnology - SARS-CoV-2 IgM/IgG	1
commercial	30	Roche - Elecsys Anti-SARS-CoV-2	2
commercial	31	Roche - Elecsys Anti-SARS-CoV-2 S	3
commercial	32	Seramun Diagnostica GmbH - SeraSpot Anti-SARS-CoV-2 IgA	1
commercial	33	Seramun Diagnostica GmbH - SeraSpot Anti-SARS-CoV-2 IgG	1
commercial	34	Serion diagnostics - Serion Elisa agile SARS-CoV-2 IgA	1
commercial	35	Serion diagnostics - Serion Elisa agile SARS-CoV-2 IgG	1
commercial	36	Siemens - Atellica IM Analyzer - SARS-CoV-2 IgG (sCOVG)	1
commercial	37	TestLine Clinical Diagnostics - EIA COVID-19 RBD IgG	1
commercial	38	Vircell microbiologists - COVID-19 ELISA IgG	2
commercial	39	Vircell microbiologists - COVID-19 ELISA IgM+IgA	3
commercial	40	Vircell microbiologists - COVID-19 VIRCLIA IgG	1
commercial	41	Zhejiang Orient Gene Biotech Co - COVID-19 IgG/IgM Rapid Test	1
In-house	42	ELISA	2
In-house	43	ELISA SARS-CoV-2 IgG	1
In-house	44	ELISA SARS-CoV-2 IgM	1
In-house	45	IFA	1
In-house	46	L3 based NT assay	1
In-house	47	Luminex multiplex immunoassay	1 laboratory (3 different variations of the test)
In-house	48	Protein micro-array	1
In-house	49	PRNT	4
In-house	50	Pseudo-neutralisation assay	1
In-house	51	surrogate VNT	1
In-house	52	suspension immunoassay based on S1 including RBD	1
In-house	53	virus microneutralisation	12
